# Aroma Investigation of New and Standard Apple Varieties Grown at Two Altitudes Using Gas Chromatography-Mass Spectrometry Combined with Sensory Analysis

**DOI:** 10.3390/molecules25133007

**Published:** 2020-06-30

**Authors:** Giulia Chitarrini, Nikola Dordevic, Walter Guerra, Peter Robatscher, Lidia Lozano

**Affiliations:** Laimburg Research Centre, Ora (BZ), 39040 Auer, Italy; giulia.chitarrini@laimburg.it (G.C.); nikola.dordevic@laimburg.it (N.D.); walter.guerra@laimburg.it (W.G.); peter.robatscher@laimburg.it (P.R.)

**Keywords:** *Malus domestica*, flavor, sensory analysis, HS-SPME-GC-MS

## Abstract

The aromatic profile of apples constitutes important information for the characterization and description of local products. Apple flavor is determined by perception in mouth and aroma; while the first is mainly defined by sugars and organic acids, aroma is a complex mixture of many volatile organic compounds (VOCs) whose composition is often specific to the variety. Headspace-solid phase microextraction gas chromatography coupled with mass spectrometry (HS-SPME-GC-MS) allows for the detection of detailed information of volatile constituents. In this study, eleven apple varieties (Braeburn, Fuji, Gala, Golden Delicious, Coop 39-Crimson Crisp^®^, Dalinette-Choupette^®^, Fujion, CIV323-Isaaq^®^, Coop43-Juliet^®^, SQ159-Natyra^®^, UEB32642-Opal^®^) grown in two pedoclimatic locations at different altitudes in South Tyrol (Italy) (ca. 225 m and ca. 650 m a.s.l.) were investigated. Thirty-eight VOCs were identified and combined with sensory analysis results (from 11 trained panelist) to characterize the aroma of new and standard apple varieties with a special focus on pedoclimatic location differences. The study shows strong diversification of the varieties based on their VOC profiles and sensory attributes, as expected. Moreover, investigating how the pedoclimatic location at different altitudes can influence the apple aroma profile, we identified twelve VOCs involved in these differences and provided a deeper investigation on how different altitudes can influence the apple aroma composition and perceptions combining the analytical and sensory parameters.

## 1. Introduction

South Tyrol is Italy’s northernmost province, located in the heart of the Alps in a very central position in Europe, and bordering Austria, Switzerland, and the Italian provinces of Sondrio, Trento, and Belluno. The territory comprises a total area of 7400 km². Nevertheless, the region is extremely mountainous, and only a small portion supports human settlement and farming.

The area used for agricultural purposes in South Tyrol amounts to 2670 km^2^, corresponding to 36% of South Tyrol’s total area. The greater part of income in the agricultural sector is earned by fruit growers [1]. South Tyrol is the largest single apple growing region in Europe; protected in the north by the Alps and open to the south, the region has the ideal climate for excellent harvests and highest product quality. The 300 days of sun per year guarantee ripe and succulent fruits of excellent flavor.

Flavor plays a very important role in consumer choice and perception of apple freshness, and a good relationship between consumer preference and sensory characteristics has been reported [2,3,4]. Consequently, to improve quality evaluation, sensory attributes assessed by trained panels should also be considered [5]. Therefore, the apples’ aromatic profile constitutes an important information for the characterization and description of local products. Trained sensory judges are able to describe the perception of product attributes through descriptive sensory analysis; moreover, one notable strength of descriptive analysis is its ability to allow relationships between descriptive sensory and instrumental analyses [6]. Flavor is determined by perception in mouth (sweetness, acidity, or bitterness), mainly defined by sugars and organic acids, and aroma, a complex mixture of many volatile organic compounds (VOCs) whose composition is specific to the variety [7,8]. VOCs are low-molecular weight compounds derived from different biosynthetic pathways with fatty acids and amino acids as precursors [7]. In addition, the contribution of each volatile compound is dependent on its particular odor threshold and the presence of other compounds [9].

It is well known that the apple aroma profile changes during fruit maturation with a general aldehyde dominance during the first stage, moving on to an alcohol compound increase during maturation, and finally showing dominance of ester compounds at maturity [10]. Aroma is influenced not only by genetic differences and maturity but also by environmental factors [11,12,13]. Changing altitude of grow locations has an impact on temperature, humidity, pressure, ultraviolet radiation, sun exposure, wind, and geology [14]. Due to the complexity of the interactions between fruit development and environment, little information is available regarding the effects of these factors on fruit quality. Few works report a deep investigation on fruit quality and fruit aroma at different altitudes; however, this factor can be crucial for selecting orchard location towards improving fruit quality. Brat et al. showed that bananas at a comparable maturity stage cultivated at a high altitude may have had different sensory characteristics from those cultivated on a plain, particularly for the *Robusta cv*. (independent of the maturity stage) grown at a high altitude, which possessed the highest concentrations of volatile compounds [15]. In grape, Tomasi et al. reported the effect of vineyard altitude on monoterpenes and norisoprenoids [16]; consequently, differences have been reported in wine with a “bell pepper” aroma exalted in wines from higher altitudes, while wines from lower altitudes were correlated with “red fruit” aromas [17,18]. In apple, the effect of altitude has been focused on in studies of physical and chemical parameters [19,20,21], and only very few works concentrated on sensory evaluation by using consumers or trained panels, such as Charles et al., who studied sensory attributes at different locations on one single apple variety [22]. Altitudes had a reported influence on aroma compounds [13,14,23], but there was a lack of information on its influence on apple VOCs. Anyway, it seems that compared with environment, year of production, and storage time, the variety is a more important source of compositional variations [24]. Several works have been published investigating the apple cultivar aroma profile [25,26]. Compounds believed to be characteristic of specific varieties have been reported; ethyl 2-methyl butanoate, 2-methyl butyl acetate, and hexyl acetate have been attributed to Fuji apples aroma, and ethyl butanoate and ethyl 2-methyl butanoate are described as active compounds in the Elstar variety [8]; however, most of them involve one or a limited number of varieties. Interesting works have been done on apple juices, and ciders considering that the apple variety is an important factor for the aroma profile of the final product [27,28,29].

Analytical chemistry, and particularly gas-chromatography with head space solid-phase microextraction (HS-SPME-GC), is a suitable approach for the analysis of VOCs in food matrices, mainly due to its simplicity, reproducibility, selectivity, and sensitivity [30,31,32,33].

In the present work we investigated the chemical composition and sensory profile of 11 apple varieties grown in two different altitudes in South Tyrol. The dataset includes standard and new varieties still poorly investigated. The aim was to highlight the aroma profile results identifying differences between the varieties and possible differences correlated with the pedoclimatic locations.

## 2. Results and Discussion

### 2.1. Volatile Organic Compound Identification in Samples

The volatile organic compounds (VOCs) of 11 apple varieties grown in two pedoclimatic location at different altitudes (A = 225 m; B = 650 m) were investigated.

Using a headspace solid phase microextraction gas chromatography mass-spectrometry (HP-SPME-GC-MS) analysis with identification and integration of the peaks, we identified 38 compounds expressed as the peak height percentage compared with the total peak heights of identified compounds in the samples under investigation (Table S1, Supplementary Materials).

The identification level for each compound is reported in Table S1 in the Supplementary Materials; level A refers to the NIST 2017 database match, level B refers to the linear retention index (LRI) match, and level C refers to a match with the commercial standard.

The main representative class of compounds in our results were esters, with a variation between 72% and 92% of the total peak height percentage. Alcohols varied between 2% and 16% and aldehydes between 2% and 9% of the total height percentage.

Among the 20 esters reported in our study, the most abundant was hexyl acetate in all samples; it varied between 24.98% in the “Juliet^®^-Coop43” site A and 66.12% in the “Gala” site B compared with the total peak height of identified VOCs. Hexyl acetate has been mentioned to confer a green, green apple, fruity, pear, and banana odor characteristic of unripe Antei apples [34,35].

The other two esters mainly represented were butyl acetate and 2-methylbutyl acetate. The first one varied from 3.58% in “Choupette^®^-Dalinette” site A up to 19.98% in the “CIV323-Isaaq^®^” site A. It is described in the literature as contributing to the ethereal fruity, fruity, and ripe banana notes [35,36,37]; 2-methylbutyl acetate has been described as one of the most important volatile esters contributing to the characteristic apple, sweet, and banana aroma [36], and it was also reported as the most effective compound of the sensory attributes for the Royal Gala apples with a red apple perception [25]. It varied from 2.19% in “Gala” site B up to 33.67% in “Natyra^®^-SQ 159” site A. Butyl acetate and 2-methylbutyl acetate appeared to be inversely present in our varieties, suggesting that they can be inversely related and influencing a different group of apple varieties. Despite both being reported as giving sweet and banana perception [34], our results indicated that the two compounds could overall contribute to the typical apple aroma as reported in literature, adding some specific notes that diversified the varieties based on their greater or lesser presence in our samples. These results are understandable considering that we analyzed complex matrices and knowing that not all detected chemical compounds can influence flavor [38]. Moreover, the compound contribution depends on specific enzyme activity and substrate availability, odor threshold, and the presence of other compounds [8].

Looking at the other compounds belonging to the group of acids, alcohols, aldehydes, and others, they represented no more than around 27% of the apple VOC profile. Among them we found hexan-1-ol as the most abundant with a variation between 1.81% and 15.38% compared with the total height of identified VOCs, which was characteristic of the “Juliet^®^-Coop43” variety site A with 15.38% of content compared with site B with 7.57% of the same variety.

A very small relative amount of 1-methoxy-4-[(*E*)-prop-1-enyl]benzene (anethole) was found in our samples, which can give an aniseed-like typical flavor in food; the same flavor perception was given by the 1-methoxy-4-prop-2-enylbenzene (estragole) compound present in a higher amount in our whole dataset (Table S1, Supplementary Materials). It is well known that esters are the main VOCs in apple; in previous studies, a range of volatiles terpenes was also detected with the predominance of (3*E*,6*E*)-3,7,11-trimethyldodeca-1,3,6,10-tetraene (alpha-farnesene) [39]. In our results, we found alpha-farnesene influencing “Fuji”, “Fujion” and “SQ 159” varieties; the biological function of alpha-farnesene is unclear and probably is not a key odor compound in apple as previously reported [39,40], even though it has been proposed together with ester compounds as responsible for cultivar classification [36].

### 2.2. Can the Altitude Influence the VOC Profile?

Excluding the differences due to the variety, we investigated the chemical differences between the pedoclimatic locations at different altitudes using a Wilcoxon signed rank test.

**Figure 1:**
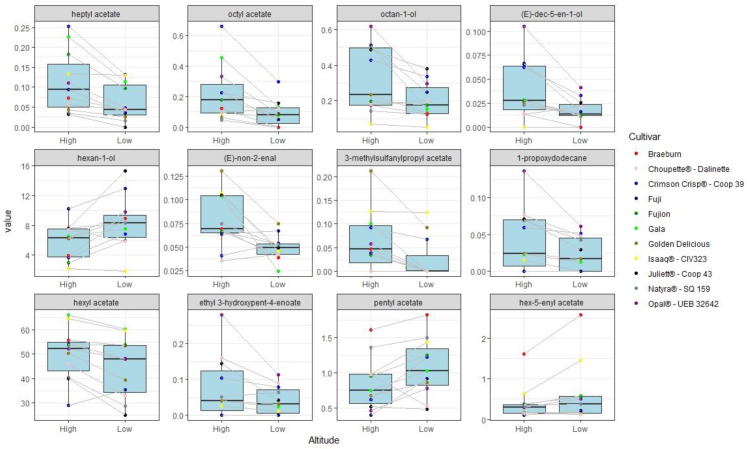
Boxplots according to statistical Wilcoxon signed rank test. Values represent the height percentage and altitude represents the different pedoclimatic locations (High = 650 m, Low = 225 m).

This test was used to order parameters according to their discriminative importance between A = low, 225 m a.s.l. and B = high, 650 m a.s.l. (Table S2, Supplementary Materials). According to statistical testing we selected the top 12 most discriminative VOCs reporting a *p* value < 0.05 (Figure 1). These compound results related to the different pedoclimatic locations independent of variety differences. Samples of the same cultivar have the same dot color connected with lines in Figure 1 to emphasize the general trend.

Heptyl acetate was the top 12 most significant compound reported, taking into account the *p* value (*p* value = 0.001) (Table S2, Supplementary Materials); it was higher in B site samples (650 m) compared with A site samples (250 m) in all varieties except for “CIV323-Isaaq^®^”, in which it seemed stable. It was reported as giving a green, citrus, waxy, and fatty characteristic [34].

Another compound affected by the geographical location with a *p* value = 0.0186 was hexyl acetate, already described as the most abundant esters we found. It was higher in B site (650 m) samples compared with A site (250 m) samples in almost all varieties except for “Coop 39-Crimson Crisp^®^” (Figure 1); in the literature, this compound is associated with green, fruity, and sweet descriptors [34].

Other compounds that were higher in B site (650 m) samples compared with A site (250 m) samples included octyl acetate (*p* value = 0.0029), reportedly giving floral, herbaceous, and fruity notes [34], and octan-1-ol (*p* value = 0.0068), described in the literature as having citrus, floral, and fatty, waxy notes [28].

By contrast, 1-hexanol appeared to be decreasing in high altitude sites compared with low altitude site in almost all varieties studied in this work except for “CIV323-Isaaq^®^” and “Choupette^®^-Dalinette” (Figure 1). Another compound significantly more present in A site samples (225 m) compared to B site samples (650 m) was acetic acid pentyl ester, which was reported as a compound giving a sensation of sweet and fruity [11,35].

### 2.3. Can the Altitude Influence Sensory Attributes and What are the Relationship between These Attributes and VOCs?

Results of two-way ANOVA on sensory data indicated significant differences on factor variety on Overall Odor, Odor-Banana, Odor-Green grass, Odor-Honey, Flavor-Pear, Flavor-Banana, and Flavor-Lemon (Table 1). Non-significant effects of the altitude factor were found on sensory attributes. However, significant interactions between factor variety and factor altitude were detected for Odor-Kiwi, Odor-Pear, Odor-Banana, Odor-Vanilla, Flavor-Lemon, Flavor-Kiwi, and Flavor-Green grass, illustrating significant effects for some combinations of altitude and variety. Similarly, Charles et al. reported honey odor perception as the only sensory attribute to have significant differences at different altitudes on the “Golden Delicious” variety [22].

Indeed, based on results of the non-parametric paired t-test, differences in the sensory attributes profile of the same variety were observed between low and high pedoclimatic locations (A-low 225 m and B-high 650 m).

Overall odor attribute was significantly different (*p* = 0.001) in the “Fuji” variety, showing more odor in the B site that in the A site.

For specifics odors attributes, Odor-Kiwi and Odor-Vanilla were significant higher (*p* = 0.034; *p* = 0.0215) in A site than in B site in the “UEB32642-Opal^®^” variety, while Odor-Banana and Odor-Honey showed significantly higher intensities (*p* = 0.0091; *p* = 0.0298) in the B site in the “Fuji” variety. In three varieties, Odor-Pear showed significant differences depending on the altitude, being higher in A site for “Gala” (*p* = 0.0243) and “SQ159-Natyra^®^” (*p* = 0.042) varieties and with higher intensity in high altitude site B for “Fujion” (*p* = 0.0108).

**Figure 2:**
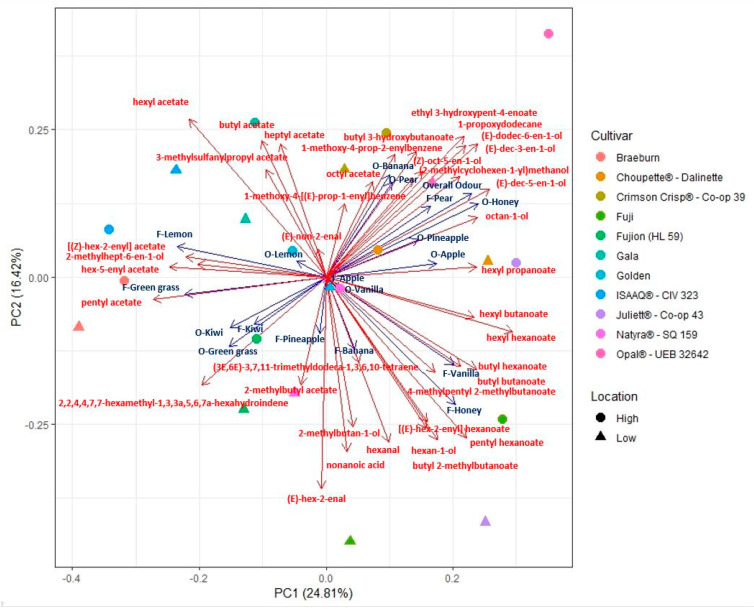
Principal component analysis reporting VOCs (red) and sensory parameters (blue) together. Varieties are reported with different colors and different shapes for low (225 m) and high (650 m) pedoclimatic locations.

The relationships between sensory parameters and VOCs were visualized using PCA (Figure 2 and Table S3 and S4, Supplementary Materials); every arrow corresponded to one parameter in the data set. the longest arrows in the direction of a particular principal component had the largest impact and importance for that PC. The same direction of groups of arrows and their lengths indicated correlation of corresponding parameters in the data set, and the opposite directed arrows indicated a negative correlation between corresponding parameters.

**Figure 3:**
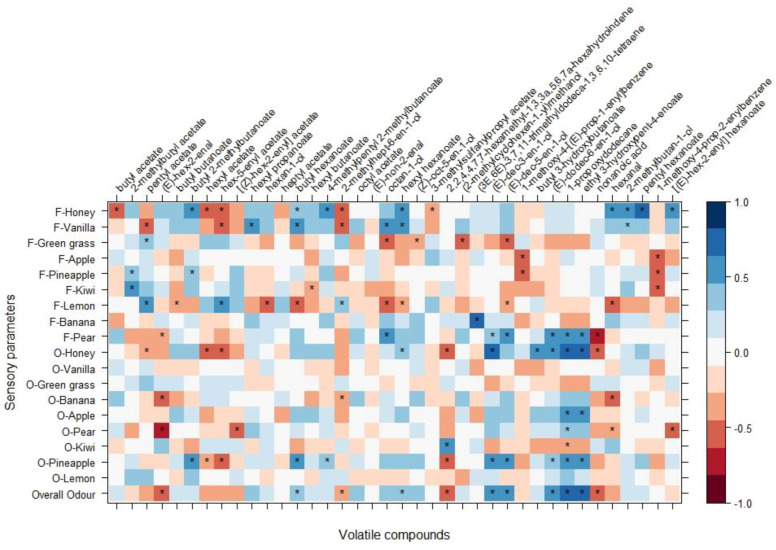
Correlation matrix. Sensory parameters and volatile organic compounds are reported as results of cross correlation analysis. Red colored squares indicate negative correlations, while blue colored squares indicate positive correlation. The significative correlations with a *p* value < 0.05 are reported with asterisks.

In addition, Spearman correlations between sensory and VOC parameters were computed. And all significant results were evaluated (Figure 3 and Table S5, Supplementary Materials).

The correlation results are visualized in the correlation matrix figure (Figure 3) in which VOCs and sensory parameters are reported. The red color indicates a negative correlation between the two corresponding parameters of the matrix, while the blue color indicates a positive correlation.

The Overall odor parameter appeared to be positively correlated with butyl hexanoate, (*E*)-dec-3-en-1-ol, (*E*)-dec-5-en-1-ol, (*E*)-dodec-6-en-1-ol, 1-propoxydodecane, and ethyl 3-hydroxypent-4-enoate (Figure 3). It was projected in the PCA with a positive influence on the first PC together with Odor-Honey, Odor-Apple, and Odor-Pineapple (Table S4, Supplementary Materials). Odor-Vanilla did not show correlation with any identified VOCs; looking at the PCA it was reported with a very short arrow in the same direction of Flavor-Vanilla, which indicates a low influence of this parameter for the PCs in our dataset. Odor-Kiwi influenced negatively the first PC (Table S4, Supplementary Materials) with a projection in PC grouped with Odor-Green grass. Odor-Banana showed only negative correlations with (*E*)-hex-2-enal, 2-methylhept-6-en-1-ol, and hexanal (Figure 3); anyhow, it was projected in the upper right quadrant of the PCA, indicating a strong relation with the other parameters influencing the PC1, such as Overall odor, Odor-Pear, and Odor-Honey (Figure 3). Odor-Honey was positively correlated with hexyl hexanoate, (*E*)-dec-3-en-1-ol, butyl 3-hydroxybutanoate, (*E*)-dodec-6-en-1-ol, 1-propoxydodecane, and ethyl 3-hydroxypent-4-enoate; and negatively correlated with pentyl acetate (fruity), hexyl acetate (described as fruity, green apple, and banana [26] and significant in our Wilcoxon signed rank test (Figure 1)), hex-5-enyl acetate, 2,2,4,4,7,7-hexamethyl-1,3,3a,5,6,7a-hexahydroindene, and nonanoic acid, which gives a fruity, green apple, banana, and waxy odor [26] (Figure 3). In the same upper right quadrant of the PCA was also located Odor-Pear, which was positively correlated with 1-propoxydodecane (Figure 3), as was Odor-Banana, and very close to Odor-Banana in the PCA (Figure 2).

Regarding specific flavor attributes, results showed significantly greater value of Flavor-Pear in higher altitude (B) in “Fuji” (*p* = 0.0243) and “Coop43-Juliet^®^” (*p* = 0.0141); Flavor-Lemon was significantly superior in B site (high altitude) in “Coop39-Crimson Crisp^®^” (*p* = 0.0165) as well as in “Fujion” (*p* = 0.008) and contrary to “SQ159-Natyra^®^” (*p* = 0.0089), which showed higher values at the low altitude location (A) (Table 1).

Flavor-Kiwi and Flavor-Green grass presented significantly higher values of intensity at high altitude (B) in the “Choupette^®^-Dalinette” (*p* = 0.0215) and “Fujion” (*p* = 0.0418) varieties (Table 1).

Both varieties also showed significantly larger values of Flavor-Pineapple in low altitudes (*p* = 0.0215, *p* = 0.0421, respectively) (Table 1). For Flavor-Honey, results revealed a bigger significant value in higher altitude locations in the “Golden Delicious” variety. Non-distinguishing significant odor and flavors were found in two varieties, “Braeburn” and “CIV323-Isaaq^®^”, in relation to the pedoclimatic location (Table S6, Supplementary Materials).

Flavor-Lemon and Flavor-Green grass were projected together influencing the PC 1 in a negative way, and they were grouped with compounds among which were pentyl acetate, hex-5-enyl acetate, [(*Z*)-hex-2-enyl] acetate, hexyl acetate, and others (Figure 2 and Table S4, Supplementary Materials). Flavor-Lemon and Flavor-Green grass results positively correlated with pentyl acetate, which was reported as a compound giving a sensation of sweet and fruity [11,27] (Figure 3). According to the PCA, we found associations between Flavor-Lemon and hex-5-enyl acetate, [(Z)-hex-2-enyl] acetate and, 2-methylhept-6-en-1-ol, with a significant positive correlation with the first and last one (Figure 2 and Figure 3).

Flavor-Honey was positively correlated with butyl 2-methylbutanoate, butyl hexanoate, 4-methylpentyl 2-methylbutanoate, hexyl hexanoate, hexanal, 2-methylbutan-1-ol, pentyl hexanoate, and [(E)-hex-2-enyl] hexanoate (Figure 3 and Table S5, Supplementary Materials). Looking at the PCA, we found a correlated group of parameters that included butyl butanoate and butyl hexanoate; and hexan-1-ol, butyl 2-methylbutanoate, [(E)-hex-2-enyl] hexanoate, pentyl hexanoate, and hexanal (Figure 2). Flavor-Honey and Flavor-Vanilla were correlated and in the same direction in the PCA, grouped with 2-methylbutan-1-ol, butyl butanoate, butyl 2-methylbutanoate, hexyl propanoate, hexan-1-ol, 4-methylpentyl 2-methylbutanoate, 2-methylhept-6-en-1-ol, hexyl hexanoate, butyl hexanoate, hexyl butanoate, [(E)-hex-2-enyl] hexanoate, and pentyl hexanoate regarding loadings (Figure 2 and Table S3, Supplementary Materials). Flavor-Honey was instead negatively correlated with butyl acetate, one of the main esters found in our results (Figure 3). Butyl acetate appeared correlated with heptyl acetate, hexyl acetate (also not significantly correlated with Flavor-Honey), and 3-methylsulfanylpropyl acetate; in fact, their arrows were in the same direction of the PCA influencing the dimension 2 of our PC (Figure 1 and Table S4, Supplementary Materials).

Flavor-Kiwi and Flavor-Pineapple had a low influence in the PC (short arrows were reported); both were significantly correlated with 2-methylbutyl acetate, already described in our experiment as one of the major esters found.

## 3. Materials and Methods

### 3.1. Apple Samples

Eleven varieties from commercial apple orchards (Malus × domestica Borkh., ca. 3000–4000 trees ha^−1^, rootstock M9, planted between 2006 and 2013) located in South Tyrol (Italy), managed according to the regional guidelines of integrated fruit production, and representing either valley (ca. 225 m a.s.l.) or hilly areas (ca. 650 m a.s.l.) were sampled during the 2014 growing season. At the valley site at the Laimburg Research Center, the soil was sandy loam with 1.7% of organic matter. In 2014, the average air temperature at 2 m was 12.8 °C with a minimum of −7.1 °C and a maximum of 34.5 °C, and 1151.8 mm yearly rainfall and 1721 yearly sunshine hours were registered [41]. At the hilly site in Vinschgau Valley, the soil was loamy sand with 5.2% organic matter. In 2014, the average air temperature at 2 m was 9.6 °C with a minimum of −10.1 °C and a maximum of 30.5 °C, and 593.3 mm yearly rainfall and 1940 yearly sunshine hours were registered. The new monogenic scab resistant varieties “Coop 39-Crimson Crisp^®^”, “Dalinette-Choupette^®^”, “Fujion”, “CIV323-Isaaq^®^”, “Coop43-Juliet^®^”, “SQ159-Natyra^®^”, and “UEB32642-Opal^®^”, which are all promising and being introduced into commercial plantings [42], and the standard varieties “Braeburn”, “Fuji”, “Gala”, “Golden Delicious” were harvested at optimal harvest stage (at a starch level between 5 and 7). Fruits were stored under normal atmosphere at 2 °C and 90% relative humidity (RH) for 60 days. VOC and sensory analysis were performed using 18 apple fruits from each of the eleven different varieties and for each of the two location (A-225 m and B-650 m) (18 fruits × 11 varieties × 2 altitudes). The eighteen fruits were selected and kept at 20 °C for 1 day prior to the analysis. Following Corollaro et al.’s method with some modification, briefly, each fruit was cut into three equal equatorial discs using an apple cutter; then, 5 to 7 cylinders (1.8 cm diameter, 1.3 cm height) per slice, depending on fruit size, were cut using a commercial apple corer (Tescoma, Brescia, Italy) [43]. Each cylinder was immediately treated with an antioxidant solution (0.2% citric acid, 0.2% ascorbic acid, 0.5% calcium chloride). Each sample was composed of 10 apple cylinders in a clear plastic cup encoded with a random three-digit code. Cylinders from the same apple fruits were used for sensory and VOC analyses.

### 3.2. Volatile Organic Compound Analysis

Volatile compounds were extracted with head space solid phase micro-extraction (HS-SPME) from the same apples used for sensory analysis. Apple cylinders (see Section 3.1) were ground with an IKA A11 analytical mill with liquid nitrogen; 2 g of fresh pulp powder were placed in 20 mL glass vials with 2 mL of milliq water, 0.75 g of NaCl, and 15 µL of 1-heptanol (15 mg/L, hexane solution) as internal standard. Following the method of Aprea et al. [44], samples were kept in agitation (250 rpm) at 40 °C for 10 min, and compounds in the headspace were captured for 30 min at 40 °C on a 2-cm DVB/CAR/PDMS 50/30 μm fiber from Supelco (Bellefonte, PA, USA). A GCMS-QP2010 SE gas chromatograph mass spectrometer (Shimadzu, Kyoto, Japan) was used to separate the compounds with a capillary ZB-WAX column (30 m × 0.25 mm i.d. × 0.25 μm) (Phenomenex, Torrance, CA, USA). The compounds were desorbed in the GC inlet at 250 °C for 5 min. The GC oven parameters were set as follow: 40 °C for 3 min, then up to 220 °C at 4 °C/min held for 1 min; then up to 250 °C at 10 °C/min held for 1 min. Helium was used as the carrier gas with a flow rate of 1.2 mL/min. The MS detector was operated in full scan mode (mass range 40–400 *m*/*z*) and the transfer line to the MS system was maintained at 250 °C. Data processing was performed using GC-MS Solution SOFTWARE (Shimadzu, Kyoto, Japan). Identification of volatile compounds was carried out by comparing mass spectra and retention indexes using the NIST 2017 database and our internal database consisting of MS spectra of commercial standards. The experimental linear temperature retention index of each compound was calculated using a series of n-alkanes (C8–C20) in the same experimental conditions as the samples. Results were expressed as peak height percentage of the individual compound compared with the total identified compounds’ height. Analysis was carried out on three technical replicates.

### 3.3. Sensory Analysis

Sensory profiling based on the quantitative descriptive analysis (QDA) method [45] was performed by a trained panel composed of eleven judges/assessors (six females and five males). All panelists were trained over 10 h (five sessions of two hours), on nineteen selected odor and flavor descriptors (Table 2). Training consisted of several types of tests of all sensory categories in order to be sure that the panelists were able to rate attributes using the linear scales anchored at the extremities with the product references (for more details on panel training and standard references refer to Corollaro et al. 2013 [43]). During sample evaluation, the panel rated odors (orthonasal perceptions by smelling) and flavors-by-mouth (retronasal perceptions by tasting) on an intensity scale going from 0 (absence) to 100 (100 maximum intensity) with graduation halfway (50). The protocols for sample preparation and testing procedure were adapted from Corollaro et al. [43]. Samples were evaluated in one session per week in four consecutive weeks, with six samples per session presented to each panelist monadically following a William’s Latin square design. Sensory evaluations were performed within 2 h of sample preparation Tests were carried out within 2 h of sample preparation in the sensory laboratory of the Laimburg Research Center equipped with individual booths and under artificial lighting.

### 3.4. Data Analysis

In this analysis, the same apples samples from 11 different varieties obtained from two different altitudes were measured in triplicate for VOC analysis, using 11 assessors for sensory analysis; missing values on one replicate of three were replaced with median values considering the other replicates for VOC analysis. To evaluate differences between the aroma profile from two different altitudes, the Wilcoxon signed-rank test was used, which considers the same apple varieties measured in two different locations. To investigate the variabilities in sensory parameters by apple variety, location, and their interaction simultaneously, a two-way repeated measures ANOVA was applied. This method takes into consideration the panelist IDs involved in the sensory panel. To assess the relationships between VOCs and sensory parameters, and climatic and geographical parameters, exploratory analysis was performed using principal component analysis (PCA) [46]. Prior to performing PCA, all parameters were normalized to 0 mean and standard deviation of 1. To evaluate the relationship between sensory and VOC parameters, Spearman correlation analysis was performed. All statistical analyses were performed using *R* software [47] with packages lattice [48] and ggplot2 [49] for the visualization and ImerTest for performing a two-way repeated measures ANOVA [50].

## 4. Conclusions

Aroma composition combined with sensory parameters of eleven apple varieties grown in two different pedoclimatic location at different altitudes in South Tyrol were investigated. The goal of this work was to analyze and characterize the aroma of new and standard apple varieties with a special focus on the differences due to the pedoclimatic locations at two different altitudes (site A = 225 m and site B = 650 m). As expected, our results show a strong differentiation on aroma profile due to the variety.

Anyhow, we were able to distinguish twelve volatile organic compounds that change in relationship with pedoclimatic locations, independent of the variety. Moreover, significant interactions were found between variety and altitude on sensory parameters. In particular, nine of the eleven varieties presented significant differences on their sensory profile, including six odors and six flavors, between low and high pedoclimatic locations. In general, we provided a deeper investigation on how different altitudes can influence the apple aroma composition and perceptions, filling the gap of available information. To the best of the authors’ knowledge, no studies have evaluated the sensory profile at low and high pedoclimatic locations by using a training panel on several apple varieties, or the relationship between these different sensory attributes and volatile organic compounds with a high number of new and commercial varieties.

From a practical point of view, this information is useful to help the growers in the site specific choice of apple varieties. On one hand, the investigated new varieties are all monogenic scab resistant, which makes them interesting for more sustainable apple growing. On the other hand, the consumer’s expectations are strongly related to the inner quality of fruits, including aroma. Anyway, further experiments would be necessary to better highlight the VOCs’ influence on aroma differentiation and consumer perception.

## Figures and Tables

**Table 1:** *p* values from two-way ANOVA on sensory data, considering variety and altitude as factors.

	Variety		Altitude		Variety * Altitude	
**Attribute**	**F Value**	***p* Value**	**F Value**	***p* Value**	**F Value**	***p* Value**
Overall Odor	8.1408	1.65 × 10^−9^	0.0019	0.9657	1.5815	0.1214
Odor-Apple	1.1746	0.3169	0.0007	0.98	0.9453	0.3169
Odor-Banana	1.9456	0.0475	0.0142	0.9074	2.9763	0.0025
Odor-Green grass	2.3315	0.0163	4.2561	0.066	1.2612	0.2627
Odor-Honey	2.1175	0.0297	0.3214	0.5833	1.1793	0.3137
Odor-Kiwi	1.8719	0.0579	0.5908	0.4599	1.9911	0.042
Odor-Lemon	1.0516	0.4068	0.3059	0.5813	1.1356	0.3427
Odor-Pear	1.5726	0.1257	0.3695	0.5568	2.4756	0.0108
Odor-Pineapple	1.6284	0.1093	0.4685	0.5092	1.0795	0.3851
Odor-Vanilla	1.1786	0.3142	2.2739	0.1625	3.2509	0.0011
Flavor-Apple	0.4536	0.9156	0.0665	0.8018	1.388	0.1967
Flavor-Banana	3.2078	0.0013	1.4151	0.2617	1.3436	0.2181
Flavor-Green grass	3.7531	0.0003	0.1727	0.6865	3.1478	0.0015
Flavor-Honey	3.7253	0.0003	0.6803	0.4287	0.6625	0.7564
Flavor-Kiwi	1.5298	0.1398	0.702	0.4217	2.5304	0.0093
Flavor-Lemon	11.259	1.12 × 10^−12^	0.3541	5.65 × 10^−1^	4.9818	7.584 × 10^−6^
Flavor-Pear	2.1496	0.0271	0.0523	0.8237	1.9076	0.0526
Flavor-Pineapple	1.4049	0.189	0.0818	0.7807	0.0818	0.1755
Flavor-Vanilla	2.4997	0.0101	1.4139	0.2619	0.5751	0.8308

**Table 2:** Sensory vocabulary used by the sensory panel.

Attributes	Definition
Overall odor	Overall odor sensation perceived via the orthonasal route
Apple	Specific odor (O) or retronasal flavor (F) apple sensation ^1^
Banana	Specific odor (O) or retronasal flavor (F) banana sensation
Green grass	Specific odor (O) or retronasal flavor (F) green grass sensation
Honey	Specific odor (O) or retronasal flavor (F) honey sensation
Kiwi	Specific odor (O) or retronasal flavor (F) kiwi sensation
Lemon	Specific odor (O) or retronasal flavor (F) lemon sensation
Pear	Specific odor (O) or retronasal flavor (F) pear sensation
Pineapple	Specific odor (O) or retronasal flavor (F) pineapple sensation
Vanilla	Specific odor (O) or retronasal flavor (F) vanilla sensation

^1^ “O” and “F“ refer to codings in Figure 2.

## Supplementary Materials

The following are available online. Table S1. VOC semi quantification in 11 apple varieties grown in two different pedoclimatic locations in South Tyrol. Results are expressed as the peak height percentage compared with the total identified peaks height. The identification level for each compound is reported: level A refers to the NIST 2017 database match, level B refers to the linear retention index (LRI) match, and level C refers to a match with an in-house pure standard. Table S2. Wilcoxon signed rank test results on VOCs. Table S3. PCA loading results. Table S4. PCA results: variable correlations with Dim1 and Dim2. Table S5. Spearman correlation results. Table S6. Wilcoxon signed rank test results on sensory parameters.
